# Convivialité des municipalités canadiennes à l’égard des aînés : portrait et facteurs associés

**DOI:** 10.17269/s41997-022-00617-9

**Published:** 2022-04-05

**Authors:** Catherine St-Pierre, Louis Braverman, Marie-France Dubois, Mélanie Levasseur

**Affiliations:** Centre de Recherche sur le Vieillissement, Centre intégré universitaire de santé et de services sociaux de l’Estrie - Centre hospitalier universitaire de Sherbrooke, Université de Sherbrooke (Québec), Sherbrooke, Québec Canada

**Keywords:** Older adults, Municipality, Age-friendly cities and communities, Active aging, Social participation, Public health policy, Aînés, municipalités, villes et communautés amies des aînés, vieillissement actif, participation sociale, politiques publiques de santé

## Abstract

**Objectives:**

This study aimed to (1) document, globally and by domain, Canadian municipalities’ level of age-friendliness, and (2) identify municipality characteristics most associated with age-friendliness.

**Methods:**

A cross-sectional survey was sent to all Canadian municipalities (*N*=3406) with an online survey of 56 items from 9 domains providing age-friendliness scores. These scores were then crossed with the following municipality characteristics: percentage of adults aged 65 and older, population density, material deprivation, social deprivation, degree of metropolitan influence, implementation step of an age-friendly municipality initiative and geographic area.

**Results:**

Nine hundred twenty-one municipalities completed the survey. Overall, municipalities’ age-friendliness total score is good (58.4%). Four domains have high scores: *Security* (80.0%)*, Respect and social inclusion* (65.0%), *Outdoor spaces and building* (62.2%), and *Social participation* (62.2%). Higher age-friendliness is associated with metropolitan municipalities, regions other than Prairies and Atlantic, higher residential density, greater proportion of older adults, greater social deprivation, lower material deprivation, and the last step of an age-friendly initiative.

**Conclusion:**

This portrait of Canadian municipalities’ age-friendliness can be used to strengthen actions promoting active aging.

## Résumé

**Objectifs:**

Cette étude visait à: 1) documenter, globalement et par domaine, le niveau de convivialité des municipalités canadiennes à l’égard des aînés, et 2) identifier les caractéristiques des municipalités les plus associées à cette convivialité.

**Méthode:**

Une enquête transversale a été soumise à l’ensemble des municipalités canadiennes (N=3 406) à l’aide d’un questionnaire en ligne composé de 56 énoncés répartis dans 9 domaines et permettant d’établir un score de convivialité à l’égard des aînés. Ces scores ont ensuite été mis en relation avec différentes caractéristiques des municipalités: pourcentage de personnes âgées de 65 ans et plus, densité de population, défavorisation matérielle, défavorisation sociale, degré d’influence métropolitaine, étape d’implantation d’une démarche Villes et communautés amies des aînés (VADA) et région géographique.

**Résultats:**

Neuf cent vingt-et-une municipalités ont complété le questionnaire. En moyenne, les municipalités présentent globalement une bonne convivialité à l’égard des aînés (58,4 %). Les quatre domaines présentant les scores les plus élevés sont : *Sécurité* (80,0 %), *Respect et inclusion sociale* (65,0 %), *Espaces extérieurs et bâtiments* (62,2 %) et *Participation au tissu social* (62,2 %). Une convivialité à l’égard des aînés supérieure est associée aux municipalités métropolitaines, aux régions autres que les Prairies et l’Atlantique, à une densité de population élevée, à une proportion supérieure d’aînés, à une défavorisation sociale supérieure, à une défavorisation matérielle inférieure et à la dernière étape d’une démarche VADA.

**Conclusion:**

Ce portrait de la convivialité des municipalités canadiennes à l’égard des aînés peut servir à renforcer les actions favorisant un vieillissement actif.

## Introduction

Portée par l’Organisation mondiale de la santé (OMS) à partir du milieu des années 2000, la démarche Villes et communautés amies des aînés (VADA) a rencontré un vif engouement partout dans le monde (Plouffe & Kalache, 2010; Fitzgerald & Caro, 2014; Moulaert & Garon, 2016; Viriot-Durandal & Scheider, 2016). Au Canada, des municipalités de toutes les provinces ont pris conscience de la nécessité de développer des environnements favorables aux aînés (Agence de la santé publique du Canada, 2016) et s’inscrivent dans cette démarche (Plouffe et al*.,* 2013). Pour ces municipalités, il s’agit de soutenir et de promouvoir un vieillissement actif de leur population.

Au-delà de la démarche VADA, plusieurs municipalités canadiennes ont également mis en place des politiques, des structures ou des services qui visent à renforcer la convivialité à l’égard des aînés. Cela renvoi à un enjeu de santé publique majeur puisqu’il s’agit, pour les municipalités, d’offrir un cadre de vie qui réponde aux besoins spécifiques d’une partie de la population, de promouvoir le bien vieillir et de prévenir les maladies. Aucune étude n’a pourtant permis de documenter le niveau de convivialité des municipalités à l’égard des aînés à l’échelle du Canada, que ce soit globalement ou selon différents domaines. Certes, des études de cas ont bien été réalisées auprès de municipalités (Garon et al*.,* 2014; Le Conseil sur le vieillissement d’Ottawa, 2017) ou au niveau de la province, notamment au Québec (Garon et al., 2016), en Colombie-Britannique (Gallagher & Mallhi, 2010) et au Manitoba (Menec et al., 2021; Menec & Nowicki, 2014; Menec et al., 2014). Avoir une meilleure vue d’ensemble de la convivialité à l’égard des aînés est cependant nécessaire pour mesurer les progrès réalisés et identifier les priorités.

Ce portrait de la convivialité des municipalités canadiennes doit aussi considérer l’influence des facteurs sociodémographiques et environnementaux. Les facilitateurs et les obstacles pour l’implantation d’actions pouvant favoriser la convivialité à l’égard des aînés diffèrent selon le type de municipalité, notamment selon l’âge de ses résidents et la densité de sa population (Menec et al., 2015a; Turcotte & Schellenberg, 2007). Au Canada, la ruralité et la disponibilité d’options alternatives de transport sont également au centre des préoccupations (Federal/Provincial/Territorial Ministers Responsible for Seniors, 2007; Menec et al., 2015b). Ainsi, afin de renforcer la cohérence des actions envers les aînés, il importe aussi d’examiner la relation entre le niveau de convivialité des municipalités à l’égard des aînés et leurs caractéristiques.

L’objectif général de cette étude est de dresser un portrait de la convivialité à l’égard des aînés offerte par les municipalités canadiennes. Plus précisément, l’étude visait à : 1) documenter, globalement et par domaine, le niveau de convivialité des municipalités à l’égard des aînés, et 2) identifier les caractéristiques des municipalités les plus associées à cette convivialité.

## Méthodes

### Dispositif

Première étape d’un programme de recherche visant à capturer comment les communautés favorisent la santé positive, la participation sociale et l’équité en santé (Levasseur et al., 2017), une enquête descriptive transversale a été soumise à l’ensemble des municipalités canadiennes, c’est-à-dire tous les territoires sur lesquels s’exercent une autorité locale conformément aux lois municipales (Commission de toponymie du Québec, 2012) et comportant un code municipal à des fins statistiques (Statistique Canada, 2015). Parmi les 3 556 municipalités canadiennes répondant à cette définition au moment de l’enquête, un total de 3 406 ont pu être contactées par courriel et ont donc été invitées à participer à un questionnaire en ligne.

Ce courriel bilingue était destiné au Directeur général de chaque municipalité et, lorsque son nom était connu, personnalisé. Il suggérait de désigner un représentant pour compléter le questionnaire en anglais ou en français : le Directeur général ou toute personne ayant une connaissance suffisante de sa municipalité. Un délai de deux semaines était accordé pour compléter le questionnaire. Dans les cas où le questionnaire n’était pas complété, deux rappels étaient effectués : le premier quatre à cinq jours avant la date limite déterminée et le second deux à trois jours après la date limite. Enfin, un nouveau courriel a été envoyé auprès des municipalités n’ayant pas complété le questionnaire à la suite du dernier rappel. Ce courriel était personnalisé et envoyé par un des collaborateurs de l’étude, soit le ministère des aînés et du logement de l’Alberta, le ministère de la santé de la Colombie-Britannique, l’Association des centres pour aînés du Manitoba ou, pour les provinces n’ayant pas un collaborateur désigné, l’Agence de la santé publique du Canada. Cette étude a obtenu l’approbation du Comité d’éthique de la recherche du CIUSSS de l’Estrie-CHUS (# 2017–656).

### Variables et outils de mesure

Le questionnaire utilisé est une version adaptée de celui conçu par Menec et collaborateurs (2015b) développé dans le cadre d’une recherche auprès de personnes âgés de 65 ans et plus sur l’initiative Communautés amies des aînés du Manitoba (Canada). Ce questionnaire est composé de 54 énoncés et présente une bonne cohérence interne (alpha de *Cronbach* variant de 0,63-0,86 selon le domaine) ainsi qu’une bonne validité apparente (Menec et al. 2015b). Pour la présente étude, s’adressant aux représentants de municipalités de toutes les provinces canadiennes et non aux Manitobains âgés, des modifications ont été apportées au questionnaire, validées par les membres de l’équipe de recherche et concernent : la suppression d’une question sur la présence de logements accessibles pour les personnes plus jeunes; l’ajout de trois énoncés, un sur l’accessibilité des arrêts de bus, un sur la présence de services communautaires à domicile abordables et un sur la disponibilité de documents officiels dans d’autres langues que le français et l’anglais. Ainsi, le questionnaire utilisé dans la présente étude comporte 56 énoncés. Afin d’éviter les opinions personnelles et d’obtenir le plus possible des réponses factuelles, quelques adaptions ont également été réalisées dans la formulation des énoncés. « À mon avis » a été remplacé par « À ma connaissance ». De même, tous les termes similaires à « est adapté », « est suffisant » ou « il y a assez » ont été remplacés par des formulations telles que « est disponible », « est offert » ou « il y a ».

Afin d’établir un score de convivialité des municipalités, le répondant était invité à répondre à 56 énoncés par « oui », « non » ou « je ne sais pas ». Ces 56 énoncés étaient répartis selon neuf domaines, le nombre d’énoncés étant variable selon le domaine (Tableau 1). Les domaines retenus sont les huit domaines prioritaires établis à la suite d’une étude internationale reposant sur le « protocole de Vancouver » (OMS, 2006, 2007) : 1) *espaces extérieurs et bâtiments*, 2) *transports*, 3) *habitat*, 4) *participation au tissu social*, 5) *respect et inclusion sociale*, 6) *participation citoyenne et emploi*, 7) *communication et information*, 8) *soutien communautaire et services de santé*. Puisqu’il s’agit d’un des piliers de base du cadre *Vieillir en restant actif* (OMS, 2002), mais aussi un élément de définition important d’une VADA, à ces huit domaines s’ajoute aussi celui de la *Sécurité*. Afin de limiter les ajouts au questionnaire, notamment pour s’assurer que les répondants veulent répondre à l’ensemble des questions de l’étude, ce domaine comporte trois énoncés qui étaient présents dans d’autres domaines du questionnaire original.

**Tableau 1:** Définitions des domaines utilisés dans le questionnaire

Domaine	Définition	Score maximum
Transports	Incluent les moyens de transport disponibles, dont les transports publics accessibles et à un coût abordable (disponibilité, coût, fréquence, fiabilité, destinations, véhicules adaptés, stationnement).	8
Habitat	Conditions de logement, qu’il s’agisse des structures, de la conception, de la situation et du choix (accessibles, bien conçus, modifications possibles, entretien disponible, variété de choix).	4
Espaces extérieurs et bâtiments	Caractéristiques du paysage urbain et de l’environnement bâti qui contribuent à rendre la ville accueillante pour les aînés (propreté, espaces verts, accessibilité, trottoirs, aires de repos, etc.).	9
Participation au tissu social	Inclut la participation à des activités récréatives, sociales, culturelles et spirituelles au sein de la communauté, et avec leur famille (accessibilité, coût, choix, intégration, information).	9
Soutien communautaire et services de santé	Disponibilité de soins de qualité, adaptés et accessibles, en quantité suffisante (accessibilité, variété, soins à domicile, soutien).	8
Communication et information	Rester en relation avec les événements et les gens et disposer d’informations pratiques pour gérer sa vie et subvenir à ses besoins (moyens de communication, accès à l’information et à la technologie).	7
Participation citoyenne et emploi	Contribution des aînés à leur communauté (bénévolat, flexibilité en emploi, formation, engagement citoyen, contribution reconnue).	4
Respect et inclusion sociale	Comportements et attitudes à l’égard des aînés (courtoisie, âgisme interactions intergénérationnelles).	4
Sécurité	Lorsque les besoins des aînés sont comblés en termes de sécurité sociale, financière et physique afin d’être protégés et de conserver leur dignité.	3
	**Score de convivialité à l’égard des aînés**	56

Le questionnaire permettait également d’identifier si la municipalité était inscrite à une démarche VADA. Les cinq jalons utilisés comme lignes directrices de l’avancement de la démarche VADA ont servi de critères pour mesurer le niveau d’implantation (Agence de la santé publique du Canada, 2016) : établir un comité consultatif, adopter une résolution par le conseil municipal, établir un plan d’action, rendre public le plan d’action, mesurer les activités. Enfin, dans une question ouverte, les répondants étaient invités à citer jusqu’à cinq priorités d’actions spécifiques au contexte de leur municipalité.

En parallèle du questionnaire, des données statistiques issues de Statistique Canada et de l’Institut national de santé publique du Québec (INSPQ) ont servi à documenter les caractéristiques des municipalités. Six variables supplémentaires ont été retenues pour l’analyse :

1. la région géographique (Colombie-Britannique, Prairies, Ontario, Québec, Atlantique et Territoires);
2. le degré d’influence métropolitaine (RMR/AR, ZIM forte, ZIM modérée, ZIM faible, aucune influence métropolitaine)^1^;
3. la proportion de résidents âgés de 65 ans et plus (exprimée en %);
4. la densité de la population, mesurée en habitants/km^2^ (densité faible = en-dessous de la valeur médiane, densité forte = égale ou au-dessus de la valeur médiane des municipalités participantes);
5. la défavorisation matérielle, exprimée en quartiles (quartile 1 = forte défavorisation, quartile 4 = faible défavorisation);
6. la défavorisation sociale, exprimée en quartiles (quartile 1 = forte défavorisation, quartile 4 = faible défavorisation)

La défavorisation matérielle reflète l’accès aux biens matériels tels que l’habitation et les transports, tandis que la défavorisation sociale reflète la fragilité du réseau social, autant au niveau de la famille que de la communauté (Pampalon et al., 2012). Les indices de défavorisation utilisés dans cette étude proviennent des données de l’Enquête nationale auprès des ménages de 2011. Puisqu’ils étaient uniquement disponibles sous forme de quintile à l’échelle des aires de diffusion (un découpage infra-municipal), une moyenne pondérée des quintiles associés à la défavorisation matérielle ou sociale composant chacune des aires de diffusion a été calculée pour chaque municipalité. Suivant la méthodologie adoptée par le CIUSSS de la Capitale-Nationale (2017), cet indice moyen permet d’ordonner chaque municipalité par rapport à toutes les municipalités canadiennes, puis de déterminer leurs quartiles.

### Analyse des données

Les répondants et les municipalités ont été décrits à l’aide de moyennes et d’écarts-types (variables continues) et de fréquences et de pourcentages (variables catégoriques). Pour le niveau de convivialité des municipalités à l’égard des aînés (objectif 1), les scores par domaine et total ont été présentés sous forme de moyenne et d’écart-type et ont également été rapportés sur 100 pour faciliter la comparaison entre les domaines à partir de pourcentages et non de scores bruts.

Pour le second objectif, à la suite de la vérification de la normalité de leur distribution et d’analyses bi-variées entre chaque caractéristique et le score total de convivialité, des analyses de régression linéaire multiple ont ensuite été réalisées pour étudier l’effet simultanée de ces caractéristiques sur le score total de convivialité. Pour les caractéristiques catégoriques, ce modèle statistique compare chaque catégorie à une de référence, par exemple, chaque région géographique par rapport au Québec. Afin de s’assurer de l’absence d’association trop forte entre deux ou plusieurs variables indépendantes, la colinéarité a été vérifiée. Tous les modèles réduits (*all possible subsets*) ont été examinés et le critère *Akaike’s Criterion Information Corrected* a guidé le choix du meilleur modèle réduit. La simplicité du modèle, le R^2^ ainsi que sa signification statistique (valeur *p* < 0,05) ont également été pris en compte. Enfin, l’analyse des résidus a été réalisée, incluant la vérification de la normalité de leur distribution à l’aide du test de Kolmogorov-Smirnov et une inspection visuelle de l’homoscédasticité. Enfin, les réponses obtenues à la question ouverte destinée à identifier les actions prioritaires ont été reclassées selon les neufs domaines explorés dans le questionnaire à partir d’une analyse de contenu thématique.

## Résultats

Un total de 921 municipalités ont répondu au questionnaire, ce qui correspond à un taux de réponse de 27,0 %.

Les municipalités répondantes et non répondantes présentaient une proportion similaire de personnes âgées de 65 ans et plus (Tableau 2). Par rapport aux municipalités non-répondantes, les municipalités répondantes appartenaient davantage aux catégories RMR/AR (27,6 vs 17,5 %). Enfin, certaines régions étaient surreprésentées parmi les municipalités répondantes (Colombie-Britannique et Québec), alors que d’autres sont sous-représentées (Prairies et Maritimes; Tableau 2).

Les personnes répondantes au questionnaire ont, en moyenne, 48 ans et possèdent onze années d’expérience. Par ailleurs, la majorité d’entre elles étaient des femmes (71,1 %), occupaient le poste de directeur général (60 %) et travaillaient dans le département de l’administration (75,8 %). La municipalités répondantes provenaient majoritairement du Québec, suivi de la Saskatchewan et de l’Ontario (Tableau 3) et se situaient dans les deux premiers quartiles de l’indice de défavorisation matérielle, qui représentent des milieux plus favorisés concernant l’accès aux biens matériels tels que l’habitation et les transports (Tableau 4). Une plus grande proportion des municipalités se situent cependant dans les derniers quartiles de l’indice de défavorisation sociale, ce qui renvoie à des milieux plus défavorisés concernant la fragilité du réseau social, autant au niveau de la famille que de la communauté. Par ailleurs, un peu moins de la moitié des municipalités avaient officiellement initié une démarche VADA (Tableau 4).

**Tableau 3:** Répartition des répondants et taux de réponse par province et territoire (*n*=921)

Province /Territoire	Fréquence (% de l’échantillon)	Taux de réponse en %
Colombie-Britannique	69 (7,5)	42,9
Québec	396 (43,0)	35,7
Île-du-Prince-Édouard^1^	25 (2,7)	34,2
Manitoba^2^	32 (3,8)	23,5
Alberta^2^	88 (9,6)	28,0
Nouveau-Brunswick^1^	29 (3,1)	27,4
Manitoba^2^	32 (3,8)	23,5
Ontario	94 (10,2)	23,0
Saskatchewan^2^	123 (13,4)	16,6
Terre-Neuve et Labrador^1^	38 (4,1)	14,5
Yukon^3^	1 (0,1)	12,5
Nunavut^3^	2 (0,2)	10,0
**Total**	921 (100,0)	27,0

^1^Regroupés sous« Atlantique » pour l’analyse par région géographique

^2^Regroupés sous « Prairies » pour l’analyse par région géographique

^3^Regroupés sous « Territoires » pour l’analyse par région géographique

**Tableau 4:** Caractéristiques des municipalités qui ont participé à l’étude

Variable	Fréquence (%)
Défavorisation matérielle (*n*=827)	
Quartile 1 Quartile 2 Quartile 3 Quartile 4	250 (30, 2) 246 (29, 7) 175 (21, 2) 156 (18, 9)
Défavorisation sociale (*n*=827)	
Quartile 1 Quartile 2 Quartile 3 Quartile 4	119 (14, 4) 207 (25, 0) 237 (28, 7) 264 (31, 9)
Étape d’implantation d’une démarche VADA (*n*=907)	
0 Aucune démarche débutée 1 Comité consultatif 2 Résolution par le conseil municipal 3 Plan d’action 4 Rendre public le plan d’action 5 Mesure des activités	489 (53,9) 55 (6,1) 36 (4,0) 73 (8,0) 39 (4,3) 215 (23,7)

**Tableau 2:** Comparaison des municipalités qui ont répondu au questionnaire avec celles qui n’ont pas participé à l’étude

	Municipalités participantes à l’étude (*n*=921)	Municipalités qui n’ont pas participées à l’étude (*n*=2610)	Valeur *p*
% 65 ans + (moyenne et é.t.)	23,34 (8,33)	23,42 (9,16)	0,84*
Densité (fréq et %)			<0,001^±^
< 42,0 habitants/km^2^ ≥ 42,0 habitants/km^2^	456 (49,8) 460 (50,2)	1539 (59,0) 1071 (41,0)	
Ruralité (ZIM) (fréq et %)			<0,001^±^
RMR/AR ZIM forte ZIM modérée ZIM faible Aucune influence	253 (27,6) 185 (20,2) 274 (29,9) 119 (13,0) 77 (8,4)	457 (17,5) 497 (19,0) 824 (31,6) 367 (14,1) 424 (16,2)	
Région géographique (fréq et %)			<0,001^±^
Colombie-Britannique Prairies Ontario Québec Maritimes Territoires	69 (7,5) 246 (26,7) 94 (10,2) 396 (43,0) 107 (11,6) 9 (1,0)	91 (3,5) 984 (37,7) 321 (12,3) 713 (27,3) 459 (17,6) 42 (1,6)	

Légende: fréq : fréquence; é.t. : écart type

^*^Valeur *p* de la comparaison entre les municipalités répondantes et les non-répondantes obtenue par Test T.

^±^Valeur *p* de la comparaison entre les répondants et les non-répondants obtenue par Test de Khi-carré.

### Niveau de convivialité des municipalités à l’égard des aînés

En moyenne, les municipalités présentent globalement une bonne convivialité à l’égard des aînés (Tableau 5). Les quatre domaines dont les scores sont les plus élevés sont : la *Sécurité*, le *Respect et inclusion sociale*, l’*Espaces extérieurs et bâtiments* et la *Participation au tissu social*. Les cinq domaines dont les scores sont les plus bas sont : le *Soutien communautaire et services de santé*, l’*Habitat*, la *Participation citoyenne et l’emploi*, la *Communication et information* et les *Transports* (Tableau 5).

**Tableau 5:** Classement des domaines de convivialité à l’égard des aînés et d’actions jugées prioritaires par les municipalités

Domaines	Scores moyens de convivialité à l’égard des aînés en % (IC à 95%; *n*=921)	Pourcentages des répondants ayant nommé une ou des actions prioritaires dans ce domaine (*n*=641) ^1^
Participation au tissu social	62,2 (60,5-63,9)	62,1
Soutien communautaire et services de santé	48,8 (47,1- 50,5)	54,9
Habitat	50,0 (47,7-52,3)	48,8
Transports	57,5 (55,8-59,2)	47,7
Espaces extérieurs et bâtiments	62,2 (60,7-63,7	42,3
Communication et information	55,7 (54,4-57,0)	19,3
Respect et inclusion sociale	65,0 (63,2-66,8)	7,6
Participation citoyenne et emploi	55,0 (52,1-56,9)	6,9
Sécurité	80,0 (78,1-81,9)	5,5
Score total	58,4 (57,3-59,5)	

Légende: IC : intervalle de confiance

^1^Parmi les 921 municipalités qui ont participé à l’étude, 641 ont répondu à une question ouverte les invitant à citer jusqu’à cinq priorités d’actions. Les municipalités répondantes ont indiqué un total de 1 891 priorités, soit 2,95 en moyenne pour chacune d’entre-elles.

### Caractéristiques des municipalités liées au niveau de convivialité

Une meilleure convivialité globale à l’égard des aînés est associée à : 1) une proportion supérieure de résidents âgés de 65 ans et plus, 2) une densité de population au-dessus ou égale à la médiane, 3) une défavorisation matérielle inférieure [Q1 vs Q4], 4) une défavorisation sociale supérieure [Q3 et Q4 vs Q1], 5) aux milieux métropolitains [RMR/AR ; comparé aux catégories ZIM fortes et Aucune influence métropolitaine], 6) aux catégories Préimplantation et Démarche complétée [comparé à Aucune démarche débutée] (Tableau 6).

**Tableau 6:** Associations entre la convivialité à l’égard des aînés et les caractéristiques des municipalités (*n*=809)

Caractéristiques	Score total
	β (SE)
% 65 ans + Valeur *p*	0,28 (0,07) <0,001
Densité de population (réf ≥ médiane)	
< médiane Valeur *p*	-9,32 (1,28) <0,001
Défavorisation matérielle (réf Q1)	
Q2 Q3 Q4 Valeur *p*	0,19 (1,25) -1,75 (1,51) -3,71 (1,73)^€^ 0,09
Défavorisation sociale (réf Q1)	
Q2 Q3 Q4 Valeur *p*	2,29 (1,63) 5,06 (1,66)^±^ 5,72 (1,78)* <0,01
Ruralité (réf RMR/AR)	
ZIM forte ZIM modérée ZIM faible Aucune infl. Valeur *p*	-6,90 (1,48)* -1,75 (1,47) -2,56 (1,72) -5,73 (2,72)^€^ <0,001
Région géographique (Réf Québec)	
Colombie-Britanique Prairies Ontario Atlantique Territoires Valeur *p*	-3,58 (1,96) -4,14 (1,64) ^±^ 0,16 (1,72) -4,95 (1,75) ^±^ 1,10 (13,46) 0,02
Démarche MADA (Réf Aucune démarche)	
Préimplantation Démarche complétée Valeur *p*	4,42 (1,26)* 8,38 (1,33)* <0,001
% variation expliquée (R^2^)	0,31

Légende : SE: standard error; * *p* < 0,001; ^±^*p* < 0,01; ^€^*p* < 0,05 lorsque comparé à la catégorie de référence

### Priorités d’actions

Parmi les 641 répondants ayant indiqué jusqu’à cinq priorités d’actions, un total de 1 891 priorités ont été identifiées et reliés à un des domaines de convivialité. Les priorités d’actions mentionnés concernaient le plus fréquemment les domaines : *Participation au tissu social*, *Soutien communautaire et services de santé*, *Habitat*, *Transports* (Tableau 5). Des exemples fréquents d’actions prioritaires étaient d’instaurer des programmes plus complets d’activités offertes aux aînés, d’offrir des logements plus abordables et d’assurer un accès aux soins de santé dans la municipalité. Les priorités d’actions les moins rapportés concernaient les domaines : *Sécurité*, *Participation citoyenne et emploi*, *Respect et inclusion sociale*.

## Discussion

La présente enquête transversale visait à documenter le niveau de convivialité des municipalités canadiennes à l’égard des aînés et à identifier les caractéristiques des municipalités les plus associées à celle-ci. Le score de convivialité à l’égard des aînés obtenu dans la présente étude est de 58,4 %, soit près de 18,4 % supérieur à celui obtenu dans la recherche de Menec & Nowicki (2014) sur les municipalités du Manitoba. Malgré l’utilisation d’un questionnaire très similaire, les municipalités sondées par Menec et Nowicki étaient surtout rurales. Or, l’accès ou la disponibilité de certains services, de politiques et de structures peuvent varier dans ces deux contextes, notamment puisque les municipalités urbaines ont généralement davantage de ressources financières pour mettre en place des actions, mais font aussi face à des contraintes bureaucratiques plus importantes (Menec et al*.*, 2014).

La présente étude montre que les domaines présentant une meilleure convivialité sont la *Sécurité*, le *Respect et inclusion sociale*, l’*Espaces extérieurs et bâtiments* et la *Participation au tissu social*. La *Sécurité* ne faisant pas officiellement partie des domaines suggérés par l’OMS pour évaluer le niveau de convivialité des villes, peu d’écrits l’ont considérée. Par ailleurs, d’autres écrits soulignent aussi l’importance des *Espaces extérieurs et bâtiments* (Green, 2013; Lehning, 2014; Menec & Nowicki, 2014; Menec et al*.*, 2014) et du *Respect et inclusion sociale* (Liddle et al., 2014; Scharlach et al*.*, 2014), deux domaines présentant un score de convivialité au-dessus de la moyenne dans la présente étude. Les résultats sont toutefois mitigés pour le domaine de la *Participation au tissu social* : en plus des présents résultats, trois études indiquent un score de convivialité important pour ce domaine (Green, 2013; Lehning, 2014; Menec et al*.,*^2014^) alors qu’une ne partage pas ce constat (Menec & Nowicki, 2014).

Comme pour les travaux de Menec & Nowicki (2014), les résultats de la présente étude montrent que le domaine de l’*Habitat* est associé à un score de convivialité au-dessous de la moyenne. Deux recherches rapportent toutefois des résultats divergents (Green, 2013; Lehning, 2014). Lehning (2014) discute d’ailleurs du fait que plusieurs municipalités urbaines offrent des incitatifs financiers pour adapter les logements aux besoins des aînés et en assurer l’accès. À l’inverse, pour les municipalités rurales étudiées par Menec et Nowicki (2014), les aînés demeurent fréquemment à domicile jusqu’à ce qu’ils soient contraints de déménager vers une municipalité où l’accès aux soins et services est plus adapté à leur situation. Enfin, en comparaison avec les présents résultats, plusieurs études ont observé une convivialité supérieure pour les *Transports* (Green, 2013; Menec & Nowicki, 2014; Lehning, 2014). Une (Lehning, 2014) de ces études avait toutefois comme répondants les agences de transport locales. Par ailleurs, les municipalités de l’étude de Green (2013) étaient toutes membres du *European Healthy Cities Network* et, ainsi, potentiellement plus impliquées dans la mise en place d’actions pour réduire les barrières à l’utilisation du transport chez les aînés.

Cette étude est la première à comparer la convivialité des municipalités à l’égard des aînés selon les régions du Canada. Le modèle statistique utilisé nous permet d’affirmer que, comparé au Québec, une moins bonne convivialité est associée aux régions de l’Atlantique et des Prairies. De plus, une convivialité à l’égard des aînés supérieure est associée aux municipalités métropolitaines, à une densité de population élevée, à une proportion supérieure d’aînés, à une défavorisation sociale supérieure, à une défavorisation matérielle inférieure et à la dernière étape d’une démarche VADA. Observée par d’autres études (Menec et al*.*, 2015b; Menec et al*.*, 2016), l’association avec une proportion supérieure d’aînés peut s’expliquer par l’hypothèse que les municipalités mettent en place un plus grande nombre d’actions favorisant un vieillissement actif lorsque la proportion d’aînés est importante. Une densité de population supérieure est également associée à une meilleure convivialité à l’égard des aînés. Wong et collègues (2015) ainsi que Wang et collaborateurs (2017) ont aussi montré que les milieux urbains possèdent une meilleure convivialité à l’égard des aînés comparativement aux ruraux. Plus largement, les défis rencontrés par les municipalités rurales pour améliorer la convivialité à l’égard des aînés sont maintenant bien connues (Menec & Novek, 2020). La présente étude propose toutefois de prendre en compte le degré d’influence métropolitaine comme variable, ce qui permet d’appréhender les territoires non pas de façon dichotomique (rural c. urbain), mais selon continuum allant des municipalités les plus rurales à celles étant davantage peuplées et qui entretiennent d’importantes relations avec un noyau urbain.

À notre connaissance, la présente étude est la première à vérifier l’influence de la défavorisation matérielle et sociale sur la convivialité des municipalités à l’égard des aînés. De manière attendue, une convivialité à l’égard des aînés supérieure est associée à une défavorisation matérielle inférieure, c’est-à-dire à un meilleur accès aux biens tels que l’habitation et les transports. Une hypothèse pouvant expliquer pourquoi la convivialité à l’égard des aînés est supérieure lorsque la défavorisation sociale est élevée est l’existence de programmes et d’initiatives, autant à l’échelle provinciale que municipale, permettant la mise en place d’actions visant à réduire les inégalités sociales et à renforcer les capacités communautaires.

Parmi ses limites, la présente étude rapporte un taux de réponse de 27,0 %. La représentativité de l’échantillon restreint partiellement la généralisation des résultats au Canada, surtout pour les municipalités rurales n’ayant aucune influence urbaine où certains éléments favorisant la convivialité à l’égard des aînés sont moins prévalents qu’en milieu urbain. Certaines régions géographiques sont surreprésentées et d’autres études sont nécessaires. Le présent portrait pourrait ainsi refléter moins fidèlement les provinces où l’on retrouve un nombre inférieur de municipalités participantes, notamment lorsqu’elles comptent un nombre élevé de municipalités rurales. Les résultats des comparaisons entre les sous-échelles, principalement celle de la sécurité qui comprend un nombre limité d’items, doivent également être interprétées de façon exploratoire et faire l’objet d’autres études. Comme tout sondage, les résultats sont sensibles au temps et au contexte des répondants et des municipalités participantes. Enfin, un biais de désirabilité sociale potentiel est lié au profil des répondants, à savoir la direction ou un représentant de la municipalité. Malgré leur bonne connaissance de la municipalité, ces répondants pourraient évaluer plus favorablement leurs politiques, leurs structures ou leurs services destinés aux personnes aînées. Le score de convivialité pourrait ainsi être supérieur à la réalité, et ce, même si les questions avaient été reformulées pour porter principalement sur des faits plutôt que des aspects subjectifs.

## Conclusion

Les aînés constituent une part importante de la population canadienne et les prévisions démographique indiquent qu’ils seront plus nombreux au cours des prochaines années. Dans ce contexte, faire des municipalités des lieux de vie conviviaux et accueillants à l’égard des aînés constitue un enjeu de santé publique majeur. Aucune recherche n’avait pourtant documenté le niveau de convivialité des municipalités à l’égard des aînés sur l’ensemble du territoire d’un pays et, plus spécifiquement, au Canada. Ces résultats peuvent ainsi nourrir les réflexions des décideurs et guider les municipalités lors de la mise en place d’actions favorisant un vieillissement actif. Cette étude attire l’attention sur certains domaines où les actions des municipalités gagnent à être développées : le *Soutien communautaire et services de santé*, l’*Habitat*, la *Participation citoyenne et l’emploi*, la *Communication et information* et les *Transports*. De même, elle invite les décideurs à prendre en considération les caractéristiques des municipalités pour ajuster les politiques destinées aux personnes aînées. Toute démarche visant à améliorer la convivialité des municipalités à l’égard des aînés mérite d’être rapportée à son contexte et d’être réfléchie en lien, par exemple, avec la densité de population ou à la proportion de résidents âgés de 65 ans et plus. D’autres études sur la convivialité des municipalités à l’égard des aînés doivent être réalisées. En plus de considérer le point de vue des aînés, tel que parfois pris en compte (Menec et al., 2015b; Wong et al., 2015; John & Gunter, 2015), ces études doivent aussi considérer l’avis d’autres acteurs clés, dont des décideurs, mais aussi des élus, des employés de la fonction publique (ex. organisateurs communautaires), incluant municipaux (ex. agent de développement rural), ou d’organismes communautaires (ex. table de concertation des aînés). En mobilisant la participation des résidents et d’autres personnes œuvrant au sein de la municipalité, il sera possible de non seulement mieux connaître les actions mises en place à l’égard des aînés, mais aussi de les évaluer et d’assurer leur pérennisation.

## Contributions à la connaissance

Qu’est-ce que cette étude ajoute aux connaissances existantes?

- Cette étude comble un déficit de connaissance sur la convivialité des municipalités à l’égard des aînés à l’échelle du Canada.
- Les indices de défavorisation matérielle et sociale sont des variables utiles à l’analyse de la convivialité des municipalités à l’égard des aînés.
- Le degré d’influence métropolitaine permet de préciser l’influence de la ruralité sur la convivialité des municipalités à l’égard des aînés.

Quelles sont les principales conséquences qu’il y a lieu d’en tirer pour les interventions, la pratique ou les politiques en santé publique?

- Les municipalités peuvent s’appuyer sur les résultats de cette étude pour mieux identifier les défis à relever et approfondir les priorités d’actions auprès des citoyens.

### Remerciements

Les auteurs tiennent à remercier Mélissa Généreux, Verena Menec, Parminder Raina, Mathieu Roy, Catherine Gabaude, Yves Couturier et Mario Paris ainsi que toutes les personnes qui ont participé à l’étude.

### Financement

Cette étude a été financée par les Instituts de recherche en santé du Canada (IRSC; # 284179). Mélanie Levasseur est une Nouvelle investigatrice des IRSC et une chercheure boursière Séniore du Fonds de la recherche en santé du Québec. Les auteurs remercient le Réseau de recherche en santé des populations du Québec (RRSPQ) pour sa contribution au financement de cette publication.

### Disponibilité des données et du matériel

Les données utilisées pour cet article sont disponibles sur demande, en contactant les auteurs.

### Disponibilité du code

Non applicable

## Footnotes

1 Les Régions métropolitaines de recensement (RMR) sont définies comme ayant une population totale d’au moins 100 000 habitants avec un noyau d’au moins 50 000 habitants et les Agglomérations de recensement (AR) comme ayant un noyau d’au moins 10 000 habitants. Les subdivisions de recensement qui ne font pas partie d’une RMR ou d’une AR sont classifiées comme des Zones d’influence métropolitaine (ZIM) forte, modérée, faible, sans influence et Territoire (Statistique Canada, 2012).

## Declarations

### Conflits d’intérêt

Les auteurs déclarent n’avoir aucun conflit d’intérêt.

### Approbation éthique et consentement à participer

Cette recherche a obtenu l’approbation du Comité d’éthique de la recherche du CIUSSS de l’Estrie-CHUS le 17 février 2017. Numéro de dossier : 2017-656.

### Consentement pour la publication

Non applicable
