# Correction to: Convivialité des municipalités canadiennes à l’égard des aînés : portrait et facteurs associés

**DOI:** 10.17269/s41997-022-00647-3

**Published:** 2022-05-05

**Authors:** Catherine St-Pierre, Louis Braverman, Marie-France Dubois, Mélanie Levasseur

**Affiliations:** Centre de Recherche sur le Vieillissement, Centre intégré universitaire de santé et de services sociaux de l’Estrie - Centre hospitalier universitaire de Sherbrooke, Université de Sherbrooke (Québec), Sherbrooke, Québec Canada

**Correction to: Canadian Journal of Public Health**

10.17269/s41997-022-00617-9

The article “Convivialité des municipalités canadiennes à l’égard des aînés : portrait et facteurs associés,“ written by Catherine St-Pierre, Louis Braverman, Marie-France Dubois, and Mélanie Levasseur, was originally published electronically on the publisher’s internet portal on April 5, 2022 without open access. With the author(s)’ decision to opt for Open Choice the copyright of the article changed on April 17, 2022 to © The Authors 2022 and the article is forthwith distributed under a Creative Commons Attribution 4.0 International License, which permits use, sharing, adaptation, distribution and reproduction in any medium or format, as long as you give appropriate credit to the original author(s) and the source, provide a link to the Creative Commons licence, and indicate if changes were made. The images or other third party material in this article are included in the article’s Creative Commons licence, unless indicated otherwise in a credit line to the material. If material is not included in the article’s Creative Commons licence and your intended use is not permitted by statutory regulation or exceeds the permitted use, you will need to obtain permission directly from the copyright holder. To view a copy of this licence, visit http://creativecommons.org/licenses/by/4.0

